# Jaundice

**Published:** 1860-12

**Authors:** 


### EDITORIAL AND MISCELLANEOUS.

### Jaundice.

Since June last, the disease heading this article has prevailed as an epidemic in Atlanta and vicinity. During the time, we have had many opportunities to study its nature, and test the efficiency of the various dissimilar plans of treatment, founded on the contradictory views of its pathology entertained by physicians.

Those who attribute the main difficulty to a suppression of the secretion of bile, of course use such means as are known usually to excite the liver to increased secretion. Among the articles for this purpose, mercury stands prominent, and is the sheet-anchor with those holding this pathological doctrine.

On the other hand counter-irritation, anodynes, alkalies, and demulcents are used by those who contend that duodenal phlogosis, or inflammation is the prime disturbance. We advocate the latter view, and feel thoroughly convinced that no permanent, or even decided benefit, has resulted from any special treatment except the application of a blister to the epigastrum. Very little has met our eye on this subject recently in Journals. From an article in the *Maryland & Virginia Med. Jour.*, by L. Faulkner, M. D., we extract the following, which looks somewhat in this direction:

"Of the pathology of jaundice, which I thought perfectly plain when I read Cullen during my pupilage, I confess my ignorance. I saw, in a case of bilious colic some years ago, the yellow color begin at a point near my cups, I think just over the liver, and spread over the body just as you have seen a shade pass along from a cloud floating across the sun, until the man was completely jaundiced, and that, too, in a few seconds. Irritation of the duodenum must be concerned in its production, and there must be some change wrought in the capillaries and other excretories by nervous influence; or the nerves may be first in this chain, and all moved by a poison in the blood. I almost regarded this epidemic as entitled to a place among eruptive diseases.

"The treatment at first, with active mercurials, I found soon to be useless, if not injurious. Indeed, I should avoid it as a poison when diphtheria was co-existing. I pushed it in one case to ptyalism, and my patient got worse and worse, although the liver seemed to pour out bile freely. It ran its course almost as some of our eruptive fevers, susceptible of guidance by mild treatment. Latterly I give only a dose or two of mercury, (calomel and bicarb. soda preferred,) with Dover's powder or opium when demanded, and then order the bowels to be kept well opened, generally with pills of castile soap, aloes and rhubarb, adding ipecac when not forbid by excessive vomiting, allowing the use of hard cider, infusing in it barberry bark in some slow cases, and a mild nutritious diet. Avoidance of wet and cold and tonics during convalescence. Counter irritation over the epigastrium was required in some cases. I tried the remedy praised by Dr. Chapman as nearer a specific than any other, but the gentleman, the same who compared as to color with his servant, took only one dose, and said he felt soap-suds foaming up in his throat all day. One other gentleman, after giving the only blue pill prepared for himself to a servant that he thought needed it more, used no other remedy than a frequent dram, for which he had an unusual appetite. Many persons, without the advice of a physician, used Castile soap in spirits I suppose, or barberry bark, upon the recommendation of older persons, who recollected the treatment when it prevailed in their young days.

P. S.—In saying that I should avoid the treatment with active mercurials as a poison when diptheria was co-existing with it, I mean the free use of this potent drug. There is nothing in regard to diptheria, this so-called new disease(?), that I am better satisfied of, than the fact that mercury is very injurious, given in repeated doses."

---

☞ The following resolutions offered by Dr. Fountain, and passed unanimously in the Scott County (Iowa) Medical Society, shows the interest now being felt by medical men, in arresting that extensive and abominable evil, criminal abortion. With the physician, to a considerable extent, rests the power to correct the depravity into which many of our race have fallen in this particular. The prompt and unqualified condemnation of abortion for convenience, as dastardly murderous, when ever the subject is hinted in the presence of physicians, would tend greatly to arrest the perpetration of this disgraceful crime:

" *Whereas*, The medical profession are everywhere cognizant of the fact that the crime of *criminal abortion* is fearfully prevalent, and increasing in all classes of society; and

" *Whereas*, The progress of civilization and the spread of religion appear not to have had the effect of diminishing this species of iniquity; therefore, be it

" *Resolved*, 1. That the members of this Society will co-operate with the American Medical Association, and other organizations of the kind, in using every effort to disseminate a knowledge of the criminal nature of practices which are too often regarded as harmless, and frequently resorted to by many who would shudder at the thought of destroying the life of a human being.

" 2. *Resolved*, That the members of this Society unite in sentiment with the opinion of the best and most learned men of the profession, in all parts of the world, that the fœtus is a living being from the earliest period of gestation, the willful destruction of which, except when required for the preservation of the life of the mother, is a crime as monstrous as infanticide, and its perpetrators should be regarded as felons by the laws of man, as they must be by every precept of morality.

"3 *Resolved*, That every member of this Society who may be known to yield to the solicitation of any party for the purposes above indicated, shall forfeit his membership, and be regarded as unworthy of fellowship by all honorable physicians.

"4. *Resolved*, That it shall be considered the duty of every physician, when application for such purpose is made, not only to decline promptly, but to exert his personal influence to the utmost, to prevent its accomplishment, by explaining its criminal character, and removing, as far as possible, the erroneous opinions, which are so generally prevalent, regarding the life of the fœtus.

"5. *Resolved*, That we denounce the common practice of newspaper proprietors in publishing advertisements which are calculated to encourage the practice of criminal abortion, as one prolific cause of a vast amount of crime and immorality, for which such newspaper editors and proprietors are thereby in a great degree responsible

"6 *Resolved*, That we likewise denounce the practice of many druggists in keeping for sale, and dispensing such preparations as are known to be used for the purpose of producing abortion, which practice is no less reprehensible than to furnish poison when knowingly purchased with murderous intent, and by which all such druggists are *participes criminis* in the evil work of corrupting good morals, and willfully engaged in aiding and assisting in the perpetration of a crime which should be held in abhorrence by every member of a civilized and Christian community."

